# Yeast display biopanning identifies human antibodies targeting glioblastoma stem-like cells

**DOI:** 10.1038/s41598-017-16066-1

**Published:** 2017-11-20

**Authors:** Michael Zorniak, Paul A. Clark, Benjamin J. Umlauf, Yongku Cho, Eric V. Shusta, John S. Kuo

**Affiliations:** 1 Neuroscience Training Program, University of Wisconsin School of Medicine and Public Health, Madison, WI 53792-8660 USA; 2 Department of Neurological Surgery, University of Wisconsin School of Medicine and Public Health, Madison, WI 53792-8660 USA; 3 Department of Chemical and Biological Engineering, University of Wisconsin School of Medicine and Public Health, Madison, WI 53792-8660 USA; 4 Carbone Cancer Center, University of Wisconsin School of Medicine and Public Health, Madison, WI 53792-8660 USA

## Abstract

Glioblastoma stem-like cells (GSC) are hypothesized to evade current therapies and cause tumor recurrence, contributing to poor patient survival. Existing cell surface markers for GSC are developed from embryonic or neural stem cell systems; however, currently available GSC markers are suboptimal in sensitivity and specificity. We hypothesized that the GSC cell surface proteome could be mined with a yeast display antibody library to reveal novel immunophenotypes. We isolated an extensive collection of antibodies that were differentially selective for GSC. A single domain antibody VH-9.7 showed selectivity for five distinct patient-derived GSC lines and visualized orthotopic GBM xenografts *in vivo* after conjugation with a near-infrared dye. These findings demonstrate a previously unexplored high-throughput strategy for GSC-selective antibody discovery, to aid in GSC isolation, diagnostic imaging, and therapeutic targeting.

## Introduction

Patients with glioblastoma (GBM) have experienced only modest improvements in survival (measured in months) after maximal surgery, radiation, temozolomide, chemotherapy and tumor-treating electrical fields^1,2^. Growing evidence suggests that tumor recurrence due to therapeutically resistant glioblastoma stem-like cells (GSC) contributes to poor survival^3–5^. Unfortunately, current markers for detection, isolation and therapeutic targeting of GSC remain scarce^6–8^ and somewhat controversial since many marker-negative tumor cells also exhibit GSC properties^9^.

Screening intact GSC cells with display libraries could identify antibodies for enriching cancer stem cells and reveal novel GSC targets for potential immunotherapeutic strategies^10^. Recent efforts were made to identify GSC targeting antibodies and peptides via phage display^11,12^ and with nucleic acid-based aptamer libraries^13^, yet cell type selectivity is still not optimal. We report an alternative approach to identify differentially binding single-chain variable fragments (scFv) and a single domain antibody (VH) via biopanning with a yeast display antibody library^14^. Cell-based screens with yeast display technology have proven successful for complexing high affinity single-chain T cell receptors (scTCR) with antigen presenting cells^15^, density centrifugation screens against mammalian lymphoid-derived cells with scTCR^16^ and biopanning to identify brain endothelial cell binding antibodies^17,18^. Beneficial to cell surface screening, multivalent display of 10^4^–10^5^ scFv on each yeast cell enhances avidity for isolation of both low affinity lead antibodies and antibodies that may recognize low abundance targets^17–19^. Moreover, the yeast display library employs a flocculin-deficient yeast strain that reduces non-specific binding to cell surfaces, thus facilitating high efficiency recovery of cell-binding scFv^17,18,20^. We therefore hypothesized that biopanning with a yeast antibody library could enrich for GSC-selective antibodies.

In this study, 6 rounds of biopanning enriched for GSC-binders, whereas subsequent positive and negative screens were used to further enhance GSC-selectivity and clonal diversity. Positive biopanning after round 6 increased the percent of recovered yeast to greater than 10%, demonstrating enrichment. Negative screens against human neural stem cells (hNSC), normal human astrocytes (NHA) and patient-matched serum-cultured GBM cells appeared to increase the observed frequency of different clones. A total of 62 unique scFv or VH clones were identified out of 598 candidates evaluated from multiple biopanning rounds in this non-saturating screen. Each unique clone was evaluated for differential binding on 12 cell lines representing human brain, patient-matched GSC and GBM cell lines. One particular clone, VH-9.7, demonstrated selectivity against all GSC lines. Flow cytometry with VH-9.7 identified human GSC from invasive orthotopic tumor xenografts. Finally, intravenously injected fluorophore-conjugated VH-9.7 detected and localized to focal GSC orthotopic xenografts. Our data successfully demonstrate a yeast biopanning approach for antibody discovery against primary human brain tumor lines, leading to identification of antibodies with potential use in research, diagnostic and therapeutic applications.

## Results

### Yeast biopanning enriches for GSC-binding scFv and VH antibodies

**Figure 1:**
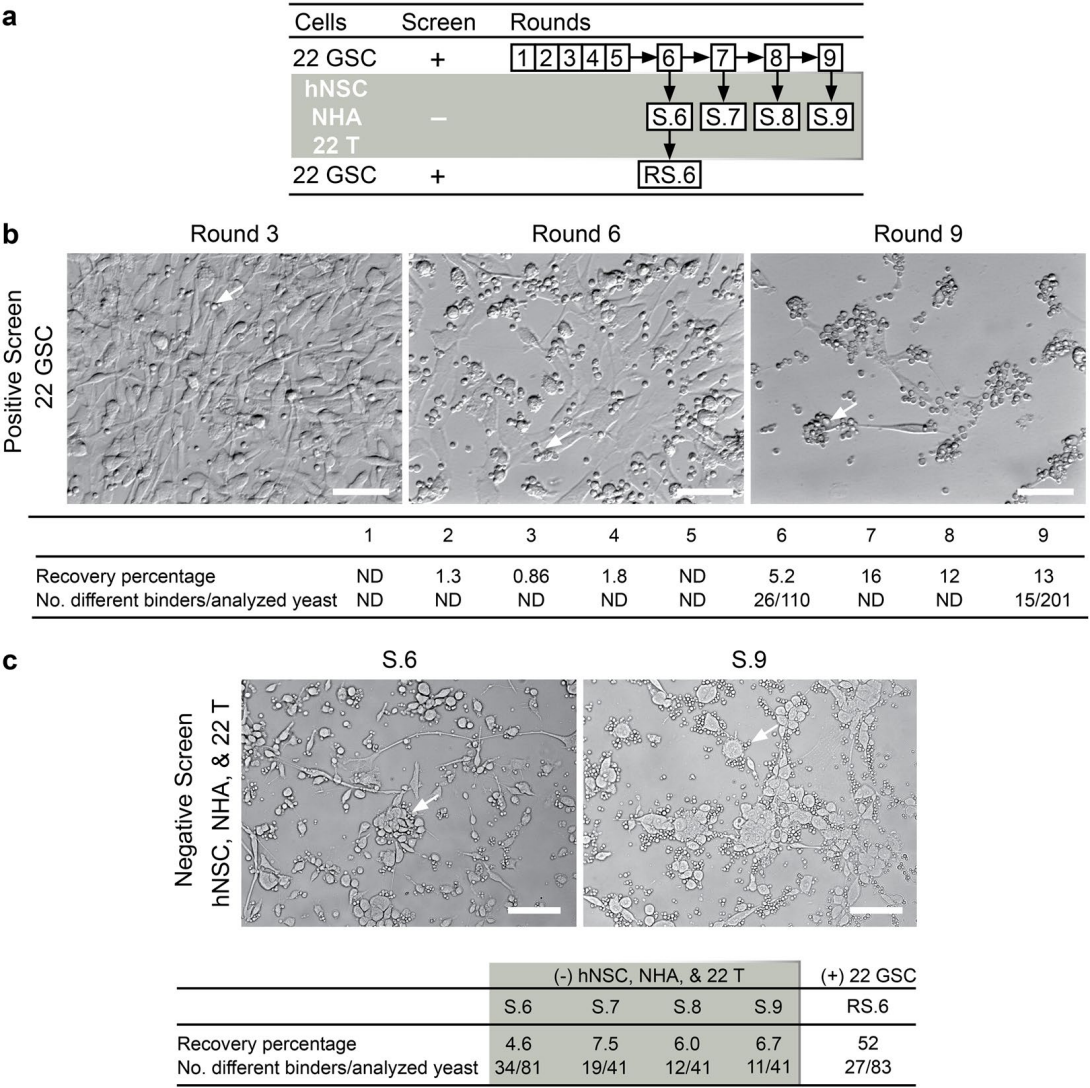
Biopanning enriches for GSC binding yeast antibodies. (**a**) Biopanning screening flow chart outlining strategy to achieve GSC-selective scFv. (**b**) A human nonimmune yeast display scFv library^18^ was screened against 22 GSC for 9 rounds and visible yeast binding to 22 GSC was observed by light microscopy. Arrows show examples of widespread yeast binding. Binding efficiency after each round of screening was evaluated by yeast plating and colony counting. An increase in the number of recovered yeast in round 6 and maintenance after round 7 indicated enrichment and screen completion, respectively. Scale bar, 50 μm. (**c**) Negative screening on hNSC, NHA, and 22 T co-culture to improve GSC-selectivity. After negative screening, photomicrographs show the depleted yeast binding after washing and recovery steps for round S.6 and S.9. Unbound yeast were recovered from each round, percentage shown. Positive re-screening on 22 GSC indicated further enrichment after depletion with 52% recovery. Scale bar, 100 µm.

The overall strategy for identification of GSC-binding scFv and VH involved enriching the yeast library against the patient-derived 22 GSC line followed by negative screening against hNSC, NHA and patient-matched serum-cultured 22 T cells (Fig. 1a). The patient-derived 22 GSC line was chosen for screening since it has been extensively characterized and generate reproducible mass-forming lesions after orthotopic implantation in the brains of non-obese diabetic severe combined immunodeficient (NOD-SCID) mice^5,21–26^. First, the yeast nonimmune human scFv library was panned against live patient-derived line 22 GSC for the identification of GSC-binders (Fig. 1b). Dissociated to single cells from spheres and seeded onto laminin overnight^27^, 22 GSC were incubated with yeast displaying scFv. GSC-binders were recovered and amplified for subsequent rounds of screening (see Methods for details), as previously described^18^. Increased binding of yeast to the GSC cell surface was microscopically observed after round 6 of biopanning (Fig. 1b) and the recovery percentage of yeast cells applied to the cell monolayer remained stable from rounds 7–9, indicating both enrichment of GSC-binding scFv and completion of the screen (Fig. 1b; Supplementary Fig. 1a). Yeast clones from round 9 demonstrated scFv-dependent binding to the GSC monolayer (Supplementary Fig. 1e). Mining a total of 311 clones from the positive screen (round 6 and round 9 pools) led to the identification of 21 unique scFv and VH by BstNI restriction digest (Supplementary Table 1, Clone ID 1–21).

To further increase antibody diversity and remove false positives, negative or subtractive yeast biopanning screening strategies were used next. To this end, a mixed co-culture system with hNSC, NHA and patient-matched serum-cultured tumor line 22 T was used to deplete 22 GSC-enriched scFv-displaying yeast pools and increase the occurrence of GSC-selective binders (Fig. 1a). Negative screens were performed by starting with pools of initial GSC-binders from biopanning rounds 6–9, and greater than 90% of initial GSC-binders were removed via depletion with the hNSC/NHA/22 T co-cultures, resulting in subtracted pools S.6–S.9 (Supplementary Fig. 1b). Since pools S.6–S.9 may still contain non-GSC-binders, a repeat positive screen on 22 GSC was performed with the S.6 pool to yield pool RS.6, which showed a 52% recovery percentage indicating high enrichment for GSC-binders (Fig. 1c; Supplementary Fig. 1c). BstNI restriction digest suggested that clone diversity was enhanced by the negative screen, since the highest number of different clones were observed in round S.6 at 34/81 (42%) (Fig. 1c; Supplementary Fig. 1b,d; Supplementary Table 1). S.6 also had 17 different clones that were not discovered in the non-saturated sampling of all unsubtracted rounds. Overall, 62 unique yeast scFv or VH clones were discovered and isolated out of a total 598 clones screened from all of the various positive and negative screening rounds (Supplementary Table 1, Clone ID 1–62).

### Evaluating cell-type binding capability of scFv and VH in yeast display format

**Figure 2:**
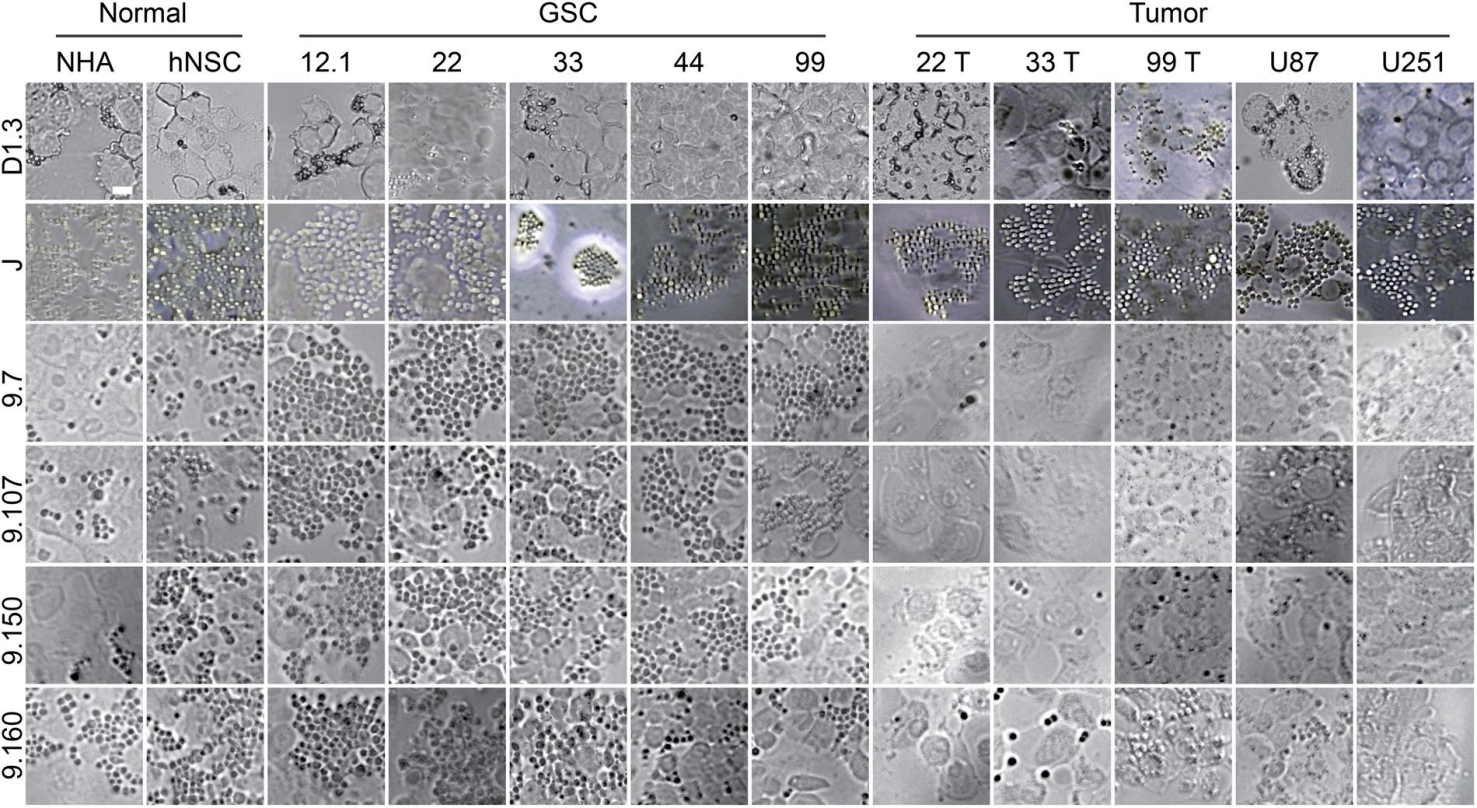
Differential GSC binding of VH-9.7, scFv-9.107, −9.150, and −9.160. Twelve different cells lines used for qualitative binding assay: normal human astrocytes (NHA); human neural stem cells (hNSC); 12.1, 22, 33, 44, & 99 GSC; and 22, 33, 99, U251, & U87 serum cultured tumor lines. Negative control: anti-lysozyme D1.3. Positive control: anti-neural cell adhesion molecule (NCAM) scFv-J. Micrographs represents one trial of three technical replicates. Scale bar, 50 μm.

To rapidly assess the cell-type binding profile of all 62 unique scFv/VH clones, each was evaluated by differential binding of the monoclonal yeast displayed scFv/VH to 12 distinct cell lines representing human brain, GSC and tumor cells with the goal of finding a GSC-selective antibody (Supplementary Fig. 3b). Each of the 5 patient-derived GSC lines have been previously clustered into classes of varying invasiveness in NOD-SCID mice according to neural lineage markers^22^. Tumor xenografts generated from 12.1 and 22 GSC lines are focal, 33 GSC are minimally invasive, whereas those derived from 44 GSC and 99 GSC are highly infiltrative. Identically numbered, standard GBM or T lines were also derived from the same patient specimens by culturing in serum-containing media, rather than sphere-forming stem cell media. Binding capability of yeast clones were determined by qualitative scoring (i.e. 0–3) of cell-bound scFv-displaying yeast (Supplementary Fig. 3a). Positive control scFv-J, anti-neural cell adhesion molecule (NCAM)^28^ bound ubiquitously to every cell line since all are derived from neural tissue. Negative control D1.3 (anti-lysozyme)^29^ maintained binding to various cell types during evaluation under manual washing conditions, suggesting presence of false positivity in this assay. Although we observed diverse scFv/VH binding from the 62 clones evaluated, yeast displayed VH-9.7 demonstrated high GSC-selectivity on 5 independent patient-derived lines and low tumor or normal brain binding (Fig. 2; Supplementary Fig. 3b). Plasmid recovery, sequencing and analysis revealed that VH-9.7 is a single-domain antibody consisting of a heavy chain of VH1 germline origin (IGHV1-46*01, IGHD3-10*01, and IGHJ4*02), without an associated light chain fragment.

In addition to VH-9.7, we identified other antibodies with similar binding profiles: scFv-9.107, 9.150, and 9.160 were GSC-selective compared with the associated patient-matched tumor lines (Fig. 2; Supplementary Fig. 3b). Also of future interest, clones isolated in subtracted pool S.6 collectively exhibited reduced binding to hNSC, NHA and tumor cell lines, while maintaining some GSC selectivity, albeit not to all 5 lines (Supplementary Fig. 3b). For instance, scFv-S.6.3 binds selectively to 33 GSC, and scFv-S.6.4 binds to most GSC except for line 22 and 99 (Supplementary Fig. 3b). In addition, from pool S.8, scFv-S.8.4 binds selectively to 33 and 99 GSC (Supplementary Fig. 3b). Many of these new clones in the subtracted pools were not found in other rounds (Supplementary Table 1). Taken together, the subtraction rounds expedited discovery of different scFv/VH clones (Supplementary Table 1) that show heterogeneous binding across different GSC lines. To our knowledge, VH-9.7 is the only VH identified from the pools of binders in this study. The remaining clones are scFv format antibodies, some showing differential GSC binding. Since VH-9.7 exhibited binding specificity against all tested GSC lines, we pursued studies with this clone for additional *in vitro* and *in vivo* experiments.

### Purified VH-9.7 retains GSC-selectivity

**Figure 3:**
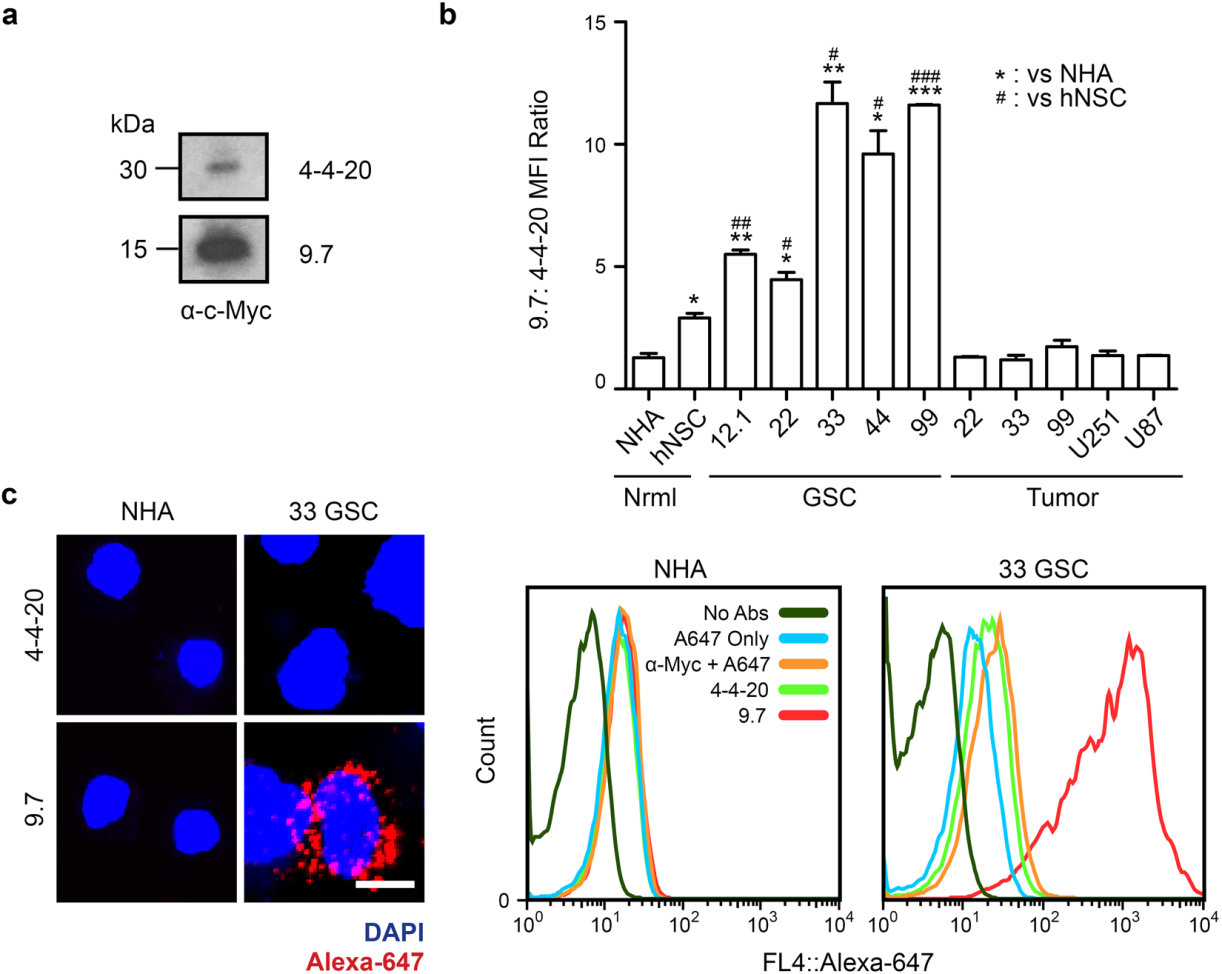
Soluble VH-9.7 is GSC-selective. (**a**) VH-9.7 was cloned into a pRS316-GAL yeast secretion vector and transformed into YVH10 yeast. Purified by Ni-NTA via a His6 tag and visualized by western blotting with anti-c-Myc. (**b**) Flow cytometry with VH-9.7 (125 nM), anti-c-Myc (66 nM), and Alexa-647. Mean fluorescence intensity (MFI) of VH-9.7 was normalized to signal generated from scFv-4-4-20. Histograms represent one experimental replicate. Two tailed unpaired t-tests used to confirm significance from two technical replicates (*^/#^p < 0.05, **^/##^p < 0.01, ***^/###^p < 0.001) versus NHA (*) and hNSC (^#^). Error bars indicate S.E.M. (**c**) Confocal microscopy performed after flow cytometry with live unfixed cells for internal consistency. Images were compiled as a z-stack projection to show cell surface labeling. Scale bar, 2.5 μm.

VH-9.7 and the negative control, anti-fluorescein scFv-4-4-20, were secreted from yeast^30^ and purified by Ni-NTA chromatography (Fig. 3a). VH-9.7 is produced predominantly in a monomeric form and has a GSC binding affinity of 74.30 ± 9.85 nM (Supplementary Fig. 4). GSC-selectivity of VH-9.7 was evaluated by flow cytometry, and binding was significantly higher (p < 0.05) to all five GSC lines compared to normal and patient-matched, serum-cultured GBM tumor cell lines (Fig. 3b). As predicted by the qualitative yeast binding assay (Fig. 2), VH-9.7 binding to NHA was not observed, while hNSC labeling by VH-9.7 was detected at a significantly lower level than VH-9.7 binding of GSC (Fig. 3b). Confocal microscopy showed punctate VH-9.7 binding to an extracellular epitope on live unfixed 33 GSC (Fig. 3c).

### Purified VH-9.7 identifies human GSC in orthotopic mouse xenografts

**Figure 4:**
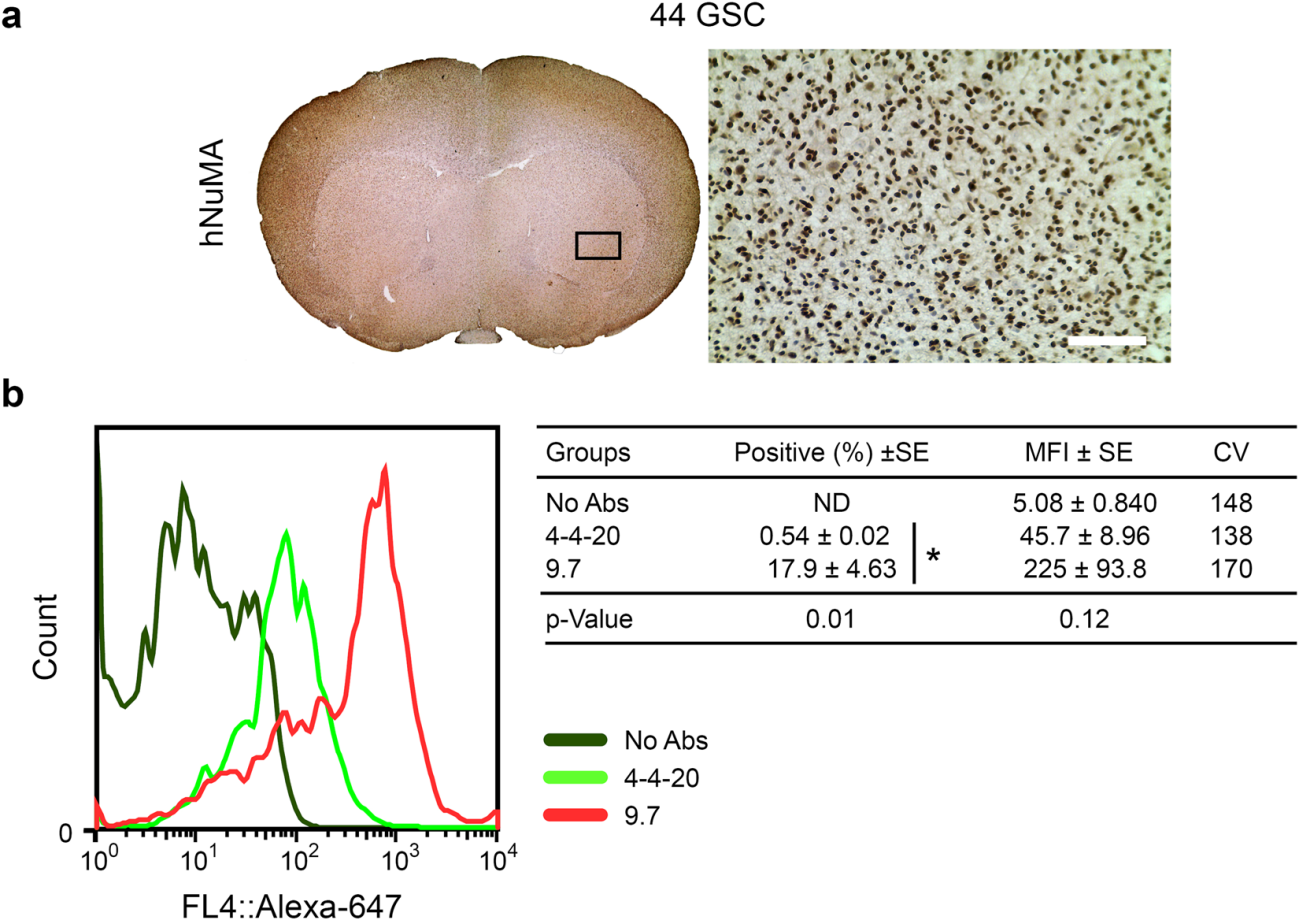
Purified VH-9.7 identifies human 44 GSC from orthotopic tumor xenografts. (**a**) Mouse brain coronal section shows whole-brain invasion of 44 GSC. Human nuclear mitotic apparatus protein (hNUMA) is specific to human cell nuclei (brown). Mouse nuclei counterstained with hemotoxylin (light blue). Black box indicates where the light micrograph was captured. Scale bar, 50 μm. (**b**) VH-9.7 specifically detects human cells from mouse brain homogenates (n = 6). No mean fluorescence intensity (MFI) signal difference was observed from normal mouse brain compared to negative control scFv-4-4-20 labeling. Histogram represents one trial of three technical replicates.

Next, we used soluble VH-9.7 to identify 44 GSC harvested from NOD-SCID mouse brain xenografts via flow cytometry. Orthotopic 44 GSC invasive tumor xenografts infiltrated throughout the entire mouse brain, shown by the diffuse distribution of cells expressing human specific nuclear mitotic apparatus protein (hNuMA) (Fig. 4a). VH-9.7 positively identified a distinct population of cells from 44 GSC xenografts (17.9% ± 4.63), whereas negative control scFv-4-4-20 did not (0.54 ± 0.02) (p = 0.01) (Fig. 4b). In additional experiments, we co-labeled the tumor/normal brain cell *ex vivo* samples with both VH-9.7 and human specific nuclear antibody (HuNu). We only observed VH-9.7 labeling (1-4%, Q2) in the human (HuNu^+^, Q1 and Q2) tumor cells, and not in normal mouse brain cells (HuNu^−^, Q3 and Q4), supporting the GSC/tumor specificity of VH-9.7 (Supplementary Fig. 5b). Additionally, the VH-9.7 antigen appeared sensitive to fixation, permeabilization, and other processing since decreased signal was observed in the co-labeling assay.

**Figure 5:**
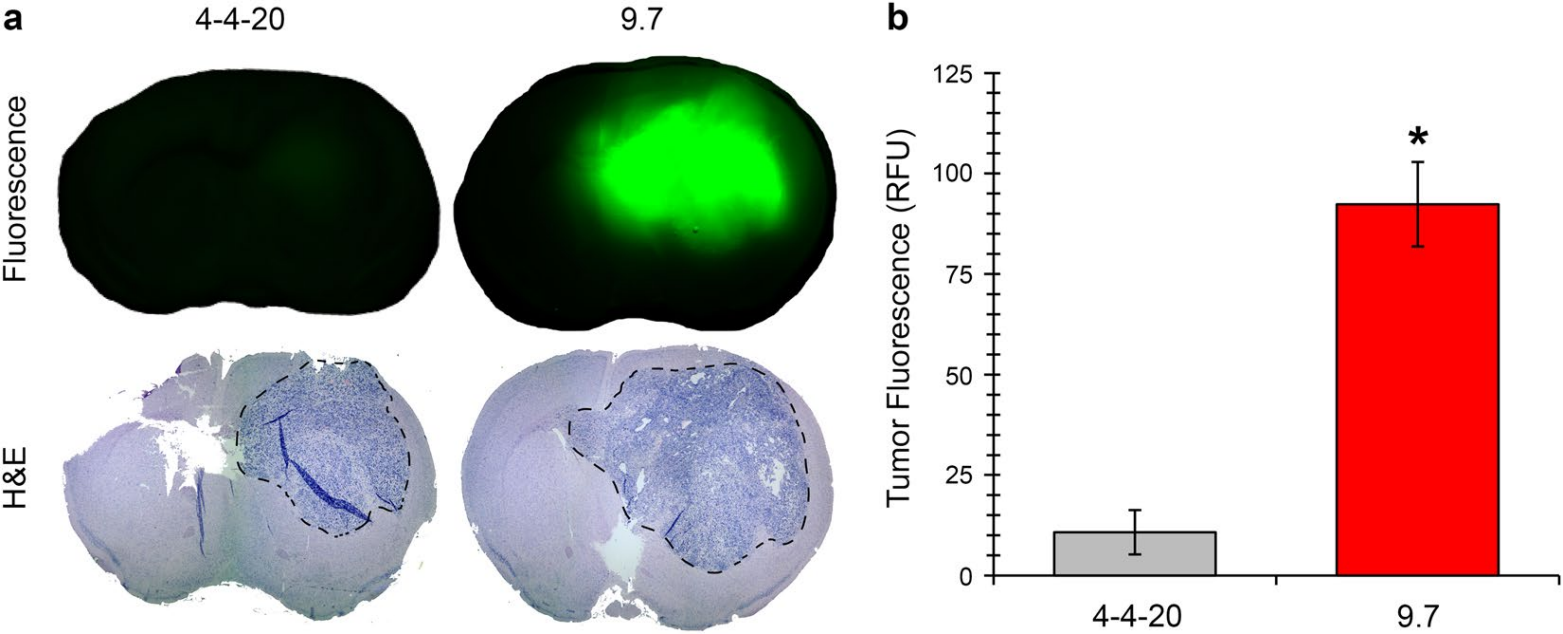
Purified near-infrared VH-9.7 localizes to human 22 GSC orthotopic xenografts. (**a**) Representative near-infrared fluorescent images captured from *ex vivo* coronal brain sections of orthotopic 22 GSC tumor xenografts in mice. Tumor area (dotted line) was identified using H&E counterstaining. (**b**) Tumor fluorescent signal was significantly higher in VH-9.7 injected mice compared to control scFv-4-4-20 (*p < 0.05, n ≥ 2 replicates, minimum of 2 independent animal experiments). Error bars indicate S.E.M. RFU: relative fluorescent units.

We chose to investigate localization of purified VH-9.7 to 22 GSC xenografts with *in vivo* infrared spectroscopy since these xenografts can be visualized with gadolinium-enhanced T1-weighted magnetic resonance imaging (data not shown). Thus, we hypothesized that the permeable tumor blood-brain barrier (BBB) of 22 GSC xenografts may permit VH entry. IgG (cetuximab), scFv-4-4-20, and VH-9.7 were mixed at a 1:1 molar ratio with IR800 dye to ensure an average of 1 fluorescent molecule for each IgG, scFv, or VH; 300 pmol of IRDye 800CW (IR800) dye with corresponding amount of IgG, scFv, and VH protein were injected intravenously into mice harboring orthotopic 22 GSC xenografts. Post-injection, an optimized time of 30 minutes was allowed to elapse for removal of unbound IR800 dye from normal tissues; additionally, transcardiac PBS perfusion further removed IR800 and unbound IgG/scFv/VH. The absence of free dye accumulation in tumor xenografts (Fig. 5; Supplementary Fig. 6) demonstrates that tumor accumulation of VH-9.7, compared to the scFv-4-4-20 or IgG (cetuximab) controls, is not an artefactual size-dependent phenomenon but due to specific VH-9.7 binding to tumor antigen. After background correction, VH-9.7 tumor specificity was quantified and fluorescent signal was substantially higher (~10-fold) than control scFv-4-4-20 (Fig. 5; Supplementary Fig. 6) (p < 0.05; n ≥ 2 replicates with minimum of 2 independent animal experiments; VH-9.7 = 92.4 ± 10.5 RFU and scFv-4-4-20 = 10.8 ± 5.51 RFU; values are average ± S.E.M.; RFU: relative fluorescent unit). Low tumor localization signal of the anti-EGFR antibody, cetuximab, is likely consistent with the previously reported low EGFR expression in 22 GSC xenografts^22^ (Supplementary Fig. 6).

## Discussion

We have isolated 62 unique scFv or VH antibodies via biopanning a nonimmune yeast antibody library against patient-derived GSC, and they show differential binding against multiple human normal, tumor, and GSC lines. Negative screening strategies increased the diversity of a randomly sampled subset of antibodies retrieved after multiple rounds of positive selection, resulting in a higher likelihood of discovering GSC-binding clones. An identified human GSC-selective VH-9.7 antibody was validated for use as a research tool to identify and enrich for GSC from orthotopic tumor xenografts, and also as an *in vivo* immunodiagnostic tool to visualize tumor xenografts. Microscopically detectable yeast expressing human antibodies have practical utility in high-throughput discovery of cell type-selective scFv or VH clones.

Four recent studies have explored phage display screening approaches against GSC. Zhu *et al*.^11^, used phage display flow cytometric sorting of CD133^+^ GBM spheres cells that were not fully validated for stem cell or tumor initiation properties. An internalizing scFv was identified, which showed some therapeutic effect when cloned into a full-length human IgG1 format and evaluated in a sphere forming assay. In Liu *et al*.^31^, a phage display peptide library was used to identify new markers of glioblastoma initiating cells *in vivo*, but selective targeting was not investigated. Similarly, Beck *et al*.^12^ reported using a 7-mer phage peptide library to identify a peptide that could be internalized and interact with nestin in GSC. When conjugated with a fluorescent quantum dot, this peptide localized specifically to orthotopic tumor xenografts. These studies demonstrate the promise of therapeutic and diagnostic protein-based modalities against GSC; however, selectivity to rigorously validated GSC lines with tumor initiation properties was not assessed. Another study using Cell-Systematic Evolution of Ligands by Exponential Enrichment (Cell-SELEX) or nucleic-acid based aptamers identified GSC-selective agents^13^, however, labeling of human neural stem cells and localization to orthotopic xenografts were not evaluated. Our study isolated yeast biopanning-identified human antibodies screened against multiple GSC lines (each rigorously validated for stem/progenitor marker expression, multipotent differentiation and tumor initiation properties) that demonstrate both GSC-selectivity and localization to orthotopic tumor xenografts, potentially expanding the repertoire of GSC targeting biologics.

To avoid any bias in isolating GSC with currently known markers, GSC lines were agnostically enriched from patient GBM tumors as spheres in minimal defined stem cell media for biopanning screening^22,32^. Validated for high efficiency tumor initiation after brain implantation in NOD-SCID mice, each GSC line recapitulates clinicopathological hallmark of GBM ranging from highly invasive to mass forming lesions^22^. Since all five patient-derived lines express a heterogeneous array of neural lineage markers, including low expression of CD133 in 44 and 99 GSC^22^, it was unexpected to observe broad GSC-selective labeling with the identified VH-9.7 antibody (Fig. 2; Fig. 3b). Although all tested GSC lines were positively labeled by VH-9.7, the more invasive 33, 44, and 99 GSC lines exhibited higher antigenic signal than 12.1 and 22 GSC that generate less invasive xenografts. It is also notable that the VH-9.7 antibody was identified from biopanning with the 22 GSC line. The inverse expression of CD133 and VH-9.7 antigen suggests that they are not synonymous. Importantly, direct *in vivo* labeling of tumor xenografts (Fig. 4; Fig. 5) and positive presence in multiple patient-derived GSC suggest that VH-9.7 antigen is probably not an artifact of *in vitro* cell culture. Future experiments are planned to identify the cognate antigen of VH-9.7^28^, residues involved with GSC binding specificity, and explore the tumor-initiating capacity and efficiency of VH-9.7 binding and VH-9.7 non-binding cells via serial implantation into brains of NOD-SCID mice^6^. Validation with these assays may provide a tool to identify and isolate GSC in addition to CD133, CD15, or marker-independent autofluorescence^6–8^.

Presumably due to the heterogeneity of GBM^33^ and GSC^22^, seven rounds of biopanning screening were required to observe enrichment on 22 GSC (Fig. 1b). The extensive number of rounds needed for enrichment raises the possibility of yeast gain-of-function genomic alterations that may enhance non-specific binding; however, we observed that GSC binding required appropriate induction of yeast scFv or VH expression after round 9 and is absent without induction of scFv or VH expression (Supplementary Fig. 1e). Similarly, phage antibody libraries theoretically require one round of screening^34^, yet when challenged with heterogeneous GBM sphere cells, three rounds were necessary via fluorescence-activated cell sorting (FACS)^11^.

Further, negative screens on co-cultured hNSC, NHA and 22 T demonstrated VH-9.7 binding specificity for GSC, and also resulted in a richer diversity of binders that were not observed in the pools recovered after positive screens alone. Subtractive screens with yeast antibody libraries have been reported before^35,36^; however, our cataloguing of clone discovery frequency and percent depletion in each round provide additional evidence for the benefits of this strategy. There was increased incidence of cell line-selective clones recovered in these pools (Supplementary Fig. 3b) which were not observed in the non-depleted pools. Clones scFv-S.6.3, -S.6.4, and -S.8.4 were differentially selective to various GSC classes with reduced binding to hNSC and NHA. On the other hand, we hypothesize that antibodies that had broad coverage among all GSC invasiveness classes, such as VH-9.7, scFv-3.107, scFv-3.150 and scFv-3.160, were presumably depleted by subtraction screening. Patient-specific scFv and VH clones will also be useful to continue exploring and characterizing GBM heterogeneity with respect to differential clinical survival.

*In vivo* localization of soluble VH-9.7 to focal 22 GSC xenografts was not surprising. Recent evidence suggests that systemic administration of bispecific T cell engaging scFv against EGFRvIII-positive U87 GBM xenografts resulted in up to 75% durable tumor control rates^37^. This suggests that scFv-based targeting platforms are potentially small enough to permeate through the partially disrupted tumor BBB in GBM. However, many central nervous system neoplasms exhibit heterogeneous permeability for smaller therapeutic molecules like paclitaxel and doxorubicin, with only a small fraction of lesions showing quantifiable uptake^38^.

Highly infiltrative 44 GSC-derived xenografts, which are not easily visualized by gadolinium-enhanced T1 MRI and may have a less permeable or intact tumor BBB, may not be readily localized via VH-9.7. This suggests the need for other strategies to solve the formidable challenge of imaging and targeting of highly invasive GSC^23,39–43^. Intracarotid infusion of hypertonic solutions (such as mannitol and arabinose) or high frequency ultrasound with microbubbles^44^ may be used to disrupt intact BBB and provide an opportunity to enhance scFv localization to invasive tumor cells in brain parenchyma^45,46^. Future experiments will explore the utility of VH-9.7 and other candidate antibodies for diagnostic and therapeutic targeting of GSC, including radiolabeled antibodies^47^, bispecific BBB-penetrating antibodies^42^, antibody-drug conjugates^48^, bispecific T-cell engaging antibodies^49^, and bioengineered immune cells such as chimeric antigen receptor T cells^50,51^.

In conclusion, we describe a high-throughput method for identifying cell type-selective scFv and VH antibodies. VH-9.7 was used as a research tool and immunodiagnostic to identify GSC *in vitro* and *in vivo*. Further development of the diagnostic and therapeutic potential of the discovered scFv and VH antibodies are underway to target therapeutically resistant GBM cells.

## Methods

All experiments were performed in accordance with relevant institutional guidelines and regulations.

### Cell culture

All studies were performed with approval from the University of Wisconsin-Madison Institutional Review Board (IRB 2012-0024) with informed consent obtained from patients, and with approval from the UW-Madison Animal Care and Use Committee (M02223). Marker-neutral isolation of GSC lines from surgical specimens was performed using our previously reported protocols^5,22,32,52–54^ and checked for mycoplasma contamination every 6 months (Lonza MycoAlert #LT07-218). Briefly, tumor tissue was collected directly from the University of Wisconsin Hospitals and Clinics operating room, weighed, coarsely minced with a scalpel blade, and subsequently chopped 2 × at 200 µm using a tissue chopper (Sorvall TC-2 Smith-Farquahar). Chopped tissue was directly plated in suspension or on laminin (0.01 mg/mL for 3 hours at 37 °C)^27^, and cultured in passaging medium: 70% Dulbecco Modified Eagle Medium (DMEM)-high glucose, 30% Ham’s F12, 1 × B27 supplement, 5 µg/mL heparin, penicillin-streptomycin-amphotericin (PSA), supplemented with 20 ng/ml each of human recombinant epidermal growth factor (EGF) and basic fibroblast growth factor (bFGF)^52^. Cultures were passaged approximately every 7–14 days by tissue chopping 2 × at 100 µm or detached with Accutase (Millipore) before plating onto freshly coated laminin flasks. GSC lines derived from distinct patients were numbered as follows: GSC lines 12.1, 22, 33, and 44 were cultured in suspension, whereas 99 was cultured on laminin, each number corresponds to a different anonymous patient source.

Each GSC line was validated for self-renewal by neurosphere formation, multipotency, tumor initiation, and serial implantation at high efficiency in NOD-SCID mice (The Jackson Laboratory) before experiments were performed^21,22^. Standard serum conditions were used to maintain patient-matched 22 T, 33 T, 99 T GBM tumor lines along with traditional U251, U87, NHA (DMEM, 10% fetal bovine serum, 1% antibiotics) (Invitrogen). In most studies, GSC were compared to hNSC, a kind gift from Dr. Clive Svendsen, and maintained as previously described^52^. Establishing and cryopreservation of cell cultures ranged from passage 1–10. Cells used for experiments ranged from passage 20–25.

### Growth and induction of scFv library

The nonimmune human scFv library harbored in the EBY100 yeast display strain (*GAL1-AGA1::URA3 ura3-52 trp1 leu2∆1 his3∆200 pep4::HIS2 prb1∆1.6 R can1 GAL*) was grown at 30 °C in 500 mL of SD-CAA (20.0 g/L dextrose, 6.7 g/L yeast nitrogen base, 5.0 g/L casamino acids, 10.19 g/L Na_2_HPO_4_•7H_2_O, 8.56 g/L NaH_2_PO_4_•H_2_O) plus 50 µg/mL kanamycin for 24 hours (OD_600_ ~ 1)^14,18^. Yeast at 10-fold excess of the library diversity (5 × 10^9^) were subsequently induced in 500 mL SG-CAA medium (same as SD-CAA except dextrose replaced by galactose) at 20 °C for 22 hours prior to biopanning against cell monolayers.

### Biopanning of scFv library against GSC monolayers

In suspension 22 GSC spheres were enzymatically dissociated with Accutase and seeded as single cells onto poly-L-lysine and laminin 6-well plates at a density of 10^7^ cells per well 12 hours prior to incubation with antibody-displaying yeast. At 10-fold excess of the library size (5 × 10^9^ yeast), induced yeast were washed twice with 0.01 M PBS, pH 7.4, supplemented with 1 mM CaCl_2_, 0.5 mM Mg_2_SO_4_ and 0.1% bovine serum albumin (BSA) (wash buffer) and the yeast mixture was added dropwise onto 126 cm2 of GSC cell monolayer to ensure even distribution at a density of 4 × 10^7^ yeast/cm2 for the first round of biopanning and monolayer surface area was proportionally decreased for subsequent biopanning rounds as the library diversity reduced (Supplementary Fig. 1a), described previously^18^. Since the pool diversity was greatly reduced after the 1^st^ round, the yeast panning density was lowered to 4 × 10^6^ yeast/cm2, and the GSC area was reduced to 50.2 cm2 for round 2 and 25.1 cm2 for rounds 3–9. The monolayers were incubated at 4 °C for 2 hours on a rotating platform (30 revolutions/minute) to allow antibody-displaying yeast to contact and bind the GSC surface. The washing strategy was optimized to recover a model scFv that binds to RBE4 cells with nanomolar apparent affinity^17,18^. The resulting method involved washing the GSC layers with ice cold wash buffer by gently rocking the plate 25×, rotating the plate 5×(repeated 2×), and rotating the plate 10×. The washing supernatant was removed after each step and replaced with fresh wash buffer. After the washing steps, 1 mL of wash buffer was added into each well and all cells were scraped off the plate and pooled together. The yeast/GSC cell mixture was resuspended in 5 mL kanamycin-supplemented SD-CAA and grown at 30 °C overnight to OD_600_ ~ 1 followed by SG-CAA induction for 20 hours at 20 °C for the subsequent round of biopanning. In parallel, a small fraction of the recovered cells were plated on SD-CAA-supplemented agar plates to quantify the number and fractional recovery of yeast cells after each round. Biopanning proceeded for 9 rounds to determine the extent of enrichment and percent yeast recovery. To confirm that the yeast-GSC interactions were scFv-based, pooled clones collected after round 9 were amplified overnight in SD-CAA at 30 °C, and half the culture was then induced in SG-CAA at 20 °C for 20 hours, to assess scFv-dependent binding of 22 GSC (Supplementary Fig. 1e).

### Negative screen biopanning on hNSC, NHA and 22 T co-culture

Negative or subtraction screens were performed by creating a co-culture of dissociated single cells of hNSC, NHA and 22 T seeded at 10^7^ cells per well in a 6-well poly-L-lysine and laminin coated plate overnight in minimal stem cell passaging media^52^. Yeast-displaying antibody pools resulting from biopanning rounds 6, 7, 8 and 9 were induced in SG-CAA for 20 hours at 20 °C and subjected to the abovementioned biopanning screening protocol on the co-cultured cells. However, the subtracted pools of unbound yeast were recovered after each washing step. A small aliquot of recovered yeast were plated on SD-CAA agar plates to calculate the percent depletion of the negative screen. After a diverse pool of clones was identified in negatively screened round S.6, it was later amplified in SD-CAA overnight and re-screened on 22 GSC to enrich for GSC-preferential scFv.

### Restriction digest analysis of scFv clones

Plasmids encoding the identified scFv were recovered from individual yeast clones isolated from the various screened pools using the Zymoprep yeast miniprep kit (Zymo Research) and amplified by PCR using the following primers: PNL6 Forward (5′-GTACGAGCTAAAAGTACAGTG-3′) and PNL6 Reverse (5′-TAGATACCCATACGACGTTC-3′). Subsequently, 20 µL of PCR product was used for BstNI (New England Biolabs) restriction digest at 60 °C for 14 hours. The digested products were resolved on a 2.5% high resolution agarose gel for unique scFv clone identification (Supplementary Fig. 2; Supplementary Table 1). Clones were named according to the screening round in which they were first discovered. “Different” clones represent diversity within a round. “Unique” clones represent the 62 antibodies with distinctive BstNI digestion patterns over all the sampled screening rounds.

### Differential microscopic screening and scoring of unique yeast clones

Twelve human cell lines (NHA; hNSC; 12.1, 22, 33, 44, & 99 GSC; 22, 33, & 99 T; U251, & U87) were seeded at 10^5^ cells per well in a 96-well plate overnight in their respective stem media or serum-containing media. Non-serum cultures required a laminin-coated surface for adherence. Each of the 62 unique yeast clones tested were induced in 5 mL SG-CAA at 20 °C. Clonal preparations of 10^6^ antibody-displaying yeast cells were allowed to incubate with each of the 12 cell lines in 200 µL of wash buffer for 2 hours at 4 °C on a rotating platform. Plates were gently washed three times with 100 µL of wash buffer with a multichannel pipette for uniformity. Light microscopy was then used to assess the binding capacity of the scFv yeast clones to each cell line and given a qualitative score from 0 to 3 (no binding to high binding) (Supplementary Fig. 3a). scFv-D1.3 (anti-lysozyme)^29^ and scFv-J (anti-NCAM)^28^ were used as negative and positive controls, respectively.

### VH-9.7 DNA sequencing

The VH-9.7 encoding plasmid was recovered using the Zymoprep yeast miniprep kit (Zymo Research) and sequenced with the Gal1–10 (5′-CAACAAAAAATTGTTAATATACCT-3′) and alpha terminator primers (5′-GTTACATCTACACTGTTGTTAT-3′) (UW-Madison Biotechnology Center). The sequence was then analyzed by IgBLAST (NCBI) to identify the human germline origin (Supplementary Information).

### ScFv and VH secretion and purification

The open reading frame for VH-9.7 was isolated from the PCR product used for BstNI typing by NheI-HindIII restriction digest and subcloned into an scFv yeast secretion vector (pRS316-GAL) that has been used extensively for scFv secretion^17,30^. The resultant pRS316-GAL-VH-9.7 plasmid was then transformed into YVH10, a yeast secretion strain overexpressing protein disulfide isomerase. Yeast harboring the pRS316-GAL-VH-9.7 secretion vector were grown in minimal SD medium (2% dextrose, 0.67% yeast nitrogen base) supplemented with 2 × SCAA amino acids (190 mg/L Arg, 108 mg/L Met, 52 mg/L Tyr, 290 mg/L Ile, 440 mg/L Lys, 200 mg/L Phe, 1260 mg/L Glu, 400 mg/L Asp, 480 mg/L Val, 220 mg/L Thr, 130 mg/L Gly, 20 mg/L tryptophan lacking leucine and uracil) at 30 °C for 72 hours. Subsequently, scFv and VH secretion was induced at 20 °C for 72 hours in SG-SCAA (dextrose substituted by galactose) with 1 mg/ml BSA as a non-specific carrier. For experiments requiring purified scFv and VH, Ni-NTA columns (Qiagen) were used to purify the six histidine-tagged scFv and VH from 50 mL or 1 L batches as described previously^55^. The size, purity, and secretion yields of scFv and VH were analyzed by SDS-polyacrylamide gel electrophoresis (PAGE) with a 10–20% gradient tris-glycine gels followed by Coomassie blue staining. Purified protein concentrations were estimated by comparison to a series of ovalbumin standards (45 kDa) (0.05 mg/mL for scFv-4-4-20 and 0.29 mg/mL VH-9.7). In parallel, the SDS-PAGE resolved proteins were also blotted onto polyvinylidene difluoride membranes (PVDF) (Millipore) for Western blotting. The PVDF membrane was blocked at 4 °C overnight in TBST solution (8 g/L NaCl, and 0.1% Tween-20, buffered to pH 7.6 with 20 mM Tris) supplemented with 5% nonfat milk and probed with 1 µg/mL 9E10 anti c-Myc antibody (Pierce) followed by an anti-mouse IgG horse radish peroxidase conjugate (Thermo Scientific). Immunocomplex detection was accomplished using Supersignal West Pico Substrate (Thermo Scientific) chemiluminescence with multiple time point exposures to CL-XPosure Film (Thermo Scientific) evaluated by ImageJ (NIH) software for quantification.

### Size exclusion chromatography

VH-9.7 was produced using a secretion strategy and purified using Ni-NTA resin (Roche), as previously described^15,56,57^. Eluted fractions were pooled and run over a TSKgel G3000SWXL (Tosoh) HPLC column using an Agilent 1200 HPLC system^58^. Proteins were detected by monitoring absorbance at 280 nm and collected using an automated fraction collector. A protein standard ranging from 670 – 14 kDa (Sigma) was used to generate the retention curved to determine size of eluted proteins. Fractions from an entire 9.7 size exclusion chromatography run were analyzed, via western blot, for the anti-c-Myc tag to determine peaks that contain VH-9.7.

### K_d_ analysis

Secreted VH-9.7 was harvested and purified via Ni-NTA and then size exclusion chromatography, as described above. Monomeric VH-9.7 was then concentrated (Millipore, 3000 MWCO Concentrators) and the amount of protein was calculated via BCA assay (Pierce). VH-9.7 was then diluted to 1,000 nM, using conditioned media from 33 GSC cultures. A titration series was then created using 2-fold dilutions with 33 GSC conditioned media ranging from 1,000.00-1.94 nM.

33 GSC, grown as spheroids, were harvested, Accutased (Innovative Cell Technologies) for 5-10 minutes, then broken apart by gentle trituration. Next, the cells were pelleted and re-suspended in condition media. Re-suspended 33 GSC were mixed 1:1 with the above mentioned dilution series to generate final concentrations of 500-0.97 nM. Cells are incubated with VH-9.7 for 1 hour at 37 °C, 5% CO_2_ with intermittent shaking. After labeling with VH-9.7, the cells are pelleted, cooled on ice, and washed two times, for 5 minutes each wash, using a blocking buffer containing 1.5% goat serum and 2% BSA in PBS. Presence of bound VH-9.7 was detected by incubating cells with 1:100 dilution of anti-c-Myc (9E10, BioLegend) and 1:200 dilution of goat anti-mouse AF647 (Life Technologies) for one hour on ice in blocking buffer. After incubation, cells were washed three times, five minutes per wash, then analyzed on a FACs Caliper (BD). Data were quantified using FlowJo, and fit to a standard one site binding model, M.F.I._Bound_ = (M.F.I._range_*[VH-9.7])/([VH-9.7] + K_d_), using the GraphPad Prism software suite to determine the binding affinity for monomeric VH-9.7. Data were normalized and presented as means and S.E.M. as previously described^59^.

### GSC-selectivity analysis by flow cytometry

To determine GSC-selectivity (Fig. 3b), approximately 10^6^ live cells were labeled in suspension (200 µL of flow cytometry buffer, PBS + 1% goat serum) at 125 nM of VH-9.7 for 1 hour at 4 °C, predimerized with Ms-anti-c-Myc (9E10) antibody followed by anti-mouse IgG AlexaFluor 647 (1:100) (Invitrogen) in the same conditions. Geometric mean fluorescence intensity was monitored by FACSCalibur flow cytometer (Becton Dickinson) after gating for live cells with propidium iodide and used to quantitate fractional bound ligand. Geometric mean fluorescence intensity (MFI) of scFv-4-4-20 was used to normalize VH-9.7 signal and two tailed unpaired t-tests were used to demonstrate significance (p < 0.05). Data represented as a ratio and S.E.M.

### GSC orthotopic xenograft model

Tumor initiation capacity of human GSC was verified by orthotopic xenograft as previously described^6,22,32,60^. Briefly, 22 and 44 GSC were enzymatically dissociated to single cells and varying cell numbers (10^2^–10^6^) were suspended in 5 μL of PBS. Using a Hamilton syringe, GSC were stereotactically implanted into the right striatum of anesthetized NOD-SCID mice at 0.33 μl/min at the following coordinates referenced from bregma: 0 mm antero-posterior, + 2.5 mm medio-lateral, and −3.5 mm dorso-ventral^60^. At either 3 months or onset of neurological symptoms, tumor formation was verified using magnetic resonance imaging (MRI). Mice were anesthetized, administered 10 mmol/kg of intra-peritoneal gadodiamide (Omniscan, GE Healthcare), for contrast-enhanced T1- and T2-weighted imaging in the UW-Madison small animal MRI scanner (Varian 4.7T). After MRI detection of tumor xenograft growth or when neurological symptoms were observed, implanted NOD-SCID mice were euthanized by perfusion fixation with 4% paraformaldehyde. Brains were then excised, embedded in paraffin, and processed for general histology. Human-specific nuclear mitotic apparatus protein (hNuMA) (Abcam, ab97585) immunohistochemistry was used to discriminate between mouse and human cells at a 1:100 dilution, as previously described^22^.

For flow cytometric identification of human GSC-derived tumor xenograft cells (n = 6) (Fig. 4), mice were sacrificed when moribund, perfused with PBS, and brains were removed to excise the region of tumor implantation in the striatum. Recovered GSC-mouse brain tissue was broken down to single cells, as described above with human brain tumors, and labeled with 125 nM of predimerized scFv-4-4-20 or VH-9.7 (not randomized or blinded). For experiments to determine human specificity of xenografts (n = 3) (Supplementary Fig. 5), prior to scFv labeling, Ms-anti-human nuclei (HuNu) (Millipore, MAB1281) at 1:100 was incubated for 1 hour at 4 °C in permeabilizing 0.1% triton-X-100 flow cytometry buffer. Human specific nuclei were detected with Gt-anti-Ms AlexaFluor 488 (1:100) (Invitrogen). Next, VH-9.7 (25 µg/mL) was predimerized with an AlexaFluor 647 (1:1 molar ratio) conjugated version of 9E10 (Santa Cruz Biotechnology Inc., sc40 AF647) so it would not conflict with HuNu secondary labeling of a mouse antibody and used to label brain tumor. After flow cytometry, scFv-labeled cells were counterstained with DAPI (Invitrogen, MP01306) at 300 nM in PBS for 5 minutes, mounted onto slides with ProLong Gold antifade reagent (Invitrogen, P36930), and imaged using confocal microscopy (Nikon A1RSi). Z-stack projection images were compiled to appreciate depth and coverage of VH-9.7 surface labeling. All flow cytometry was analyzed using FlowJo v10.0.6.

### Near-infrared fluorescence imaging of 22 GSC orthotopic xenografts

Near-infrared fluorescence imaging (NIRF) was performed with conjugates of cetuximab, scFv-4-4-20, and VH-9.7 with IRDye 800CW-NHS ester (LI-COR Biosciences Co.) at a 1:1 molar ratio, as previously described^61^. Briefly, 800CW-NHS dissolved in DMSO (25 mg/ml) was combined with IgG, scFv, or VH (1:1 molar ratio) in PBS (pH 8.5) for 2 hours at room temperature. The resulting solution was sterile filtered through Millipore Ultrafree Centrifugal Filters (0.65 µm). 800CW-labeled IgG, scFv, or VH were injected via the tail vein at a dose of 300 pmol per mouse harboring 22 GSC xenografts. Dorsal coats of mice were shaved and imaged at 0, 0.5, 1, and 2 hours post-injection to determine the optimal differential signal between scFv-4-4-20 and VH-9.7 with the IVIS Spectrum Pre-Clinical *In Vivo* Imaging System (Caliper Life Sciences). Under 2% isoflurane in O_2_, mice were visualized with 745 nm excitation and 800, 820, and 840 nm emission spectra, collecting radiance (photons/second/cm^2^/steradian) for approximately 1 minute at each time point. Near-infrared signal appeared highest in the animals’ right cerebral hemisphere 30 minutes after injection and decayed to baseline after 2 hours. One week later, mice were re-injected with soluble IgG, scFv, or VH, and sacrificed at 30 minutes post-injection by intracardiac PBS perfusion to remove blood-borne, unbound IgG, scFv, or VH. Brains were then harvested and fixed in 4% paraformaldehyde for 24 hours. Coronal cross-sections were made at the coordinates of tumor implantation and imaged *ex vivo* using a NIRF scanning system (800 nm channel, Odyssey, LI-COR Biosciences). Resultant fluorescent brain scans were background-corrected by subtracting mean signal of the contralateral striatum from the entire brain. Brains were then histologically processed and counterstained with hematoxylin and eosin (H&E). Tumor area was defined using H&E, and the mean fluorescent intensity of the tumor area determined using Photoshop (version CS6, Adobe Systems).

### Data availability

All relevant data are available from the authors.

## Electronic supplementary material

Supplementary Information
