# Supplementary material for: Yeast display biopanning identifies human antibodies targeting glioblastoma stem-like cells
Source: Sci Rep. 2017 Nov 20;7:15840. doi: 10.1038/s41598-017-16066-1 (PMC5696472; doi:10.1038/s41598-017-16066-1)
Supplement: Supplementary file 1 — Supplementary Information [file 41598_2017_16066_MOESM1_ESM.pdf]

Yeast display biopanning identifies human antibodies targeting glioblastoma stem-like cells

Michael Zorniak[1,2], Paul A. Clark[2], Benjamin J. Umlauf[3], Yongku Cho[3], Eric V. Shusta*[3,4], & John S. Kuo*[1,2,4]

[1]Neuroscience Training Program, [2]Department of Neurological Surgery, [3]Department of Chemical and Biological Engineering, [4]Carbone Cancer Center, University of Wisconsin School of Medicine and Public Health, Madison, WI, 53792-8660, USA. Correspondence and requests for materials should be addressed to E.V.S. (eshusta@wisc.edu) and J.S.K. (email: kuo@neurosurgery.wisc.edu).

Supplementary information

Supplementary Figure 1. Descriptive statistics of biopanning screening. (**a**) Positive screening statistics against 22 GSC. (**b**) Negative screening statistics against hNSC, NHA, and 22T co-culture. (**c**) Re-positive screening statistics against 22 GSC. (**d**) Pie charts show increase in antibody clone diversity and selectivity after negative screening. scFv-9.1 is non-specific, and VH-9.7 is GSC-selective. Notably, by round 9 nearly half of the discovered clones are non-specific scFv-9.1. Negative screening rounds S.7, S.8, and S.9 show a steady rise in non-specific scFv-9.1 from 4.88% to 24.4 %, but still less than 45.8% observed in round 9 alone. (**e**) Antibody-dependent enrichment was determined by incubating yeast binders from round nine with GSC in the presence or absence of antibody expression-inducing media (galactose) or non-inducing media (glucose). Scale bar, 100 μm.

Supplementary Figure 2. Unique clones discovered by plasmid isolation, PCR and BstNI restriction enzyme digest revealing different patterns on DNA gel electrophoresis. 62 unique scFv or VH clones were isolated out of 598 total yeast screened. Clones were isolated from round 6, 9, S.6, S.7, S.8, S.9, and RS.6. MW = Molecular Weight.

Supplementary Figure 3. Unique yeast scFv and VH screened for GSC-selectivity. (**a**) Examples of qualitative scoring of yeast scFv binding with various intensity, from 0 (no observed binding) to 3 (significant yeast binding). Yeast were seeded in 96 well plates, incubated for 2 hours at 4 ºC and washed according to the biopanning protocol[1]. Scale bar, 50 μm. (**b**) Heat map of 62 yeast scFv or VH clones binding 12 different cells lines: normal human astrocytes (NHA); human neural stem cells (hNSC); 12.1, 22, 33, 44, & 99 GSC; and 22, 33, 99, U251, & U87 serum cultured tumor lines. Negative control: anti-lysozyme D1.3. Positive control: anti-neural cell adhesion molecule (NCAM) scFv-J.

Supplementary Figure 4. Monomeric affinity (Kd) of VH-9.7 on 33 GSCs. (**a**) VH-9.7 is produced largely as monomer. Ni-NTA purified VH-9.7 was analyzed by size exclusion chromatography. Elution fractions were collected and analyzed via western blotting for VH-9.7 against the c-Myc epitope tag. The major VH-9.7 peak (retention time = 13.1 min) corresponded to a size of 15.3 kDa, and the expected monomeric size of VH-9.7 is ~15 kDa. Arrowheads represent the elution times for molecular size standards. 97% of the VH-9.7 detected by western blotting was monomeric. (**b**) Affinity titration of monomeric VH-9.7 on 33 GSCs. Monomeric VH-9.7 was titrated against 33 GSCs and binding assessed by flow cytometry. Fitting to a standard biomolecular binding equilibrium model[2], these data yielded a K_d of 74.30 ± 9.85 nM. The results of four independent titrations are plotted as mean ± SEM. (**c**) Full-length immunoblot of Figure 3a elution fractions for VH-9.7 and scFv-4-4-20. Dashed boxes indicate location of image cropping.

Supplementary Figure 5. VH-9.7 labels human cells in formalin fixed orthotopic mouse 44 GSC-derived orthotopic tumor xenografts. (**a**) Dissociated brains from mouse xenografts were labeled with Ms-anti-c-Myc:AlexaFluor 647 (66 nM) and Gt-anti-Ms-IgG:AlexaFluor 488 (1:100) to serve as a negative control. (**b**) Addition of Ms-anti-human nuclei (HuNu) (1:100) and VH-9.7 (125 nM) to three 44 GSC-derived xenografts indicating human-specific labeling of VH-9.7 in quadrant 2 (Q2).

Supplementary Figure 6. Purified near-infrared VH-9.7 localizes to human 22 GSC orthotopic xenografts. Representative near-infrared fluorescent images captured from ex vivo coronal brain sections of orthotopic 22 GSC-derived tumors in mice. Tumor area was identified using H&E counterstaining. 22 GSC-derived xenografts have low expression of EGFR and the anti-EGFR antibody cetuximab was used as a control.

VH-9.7 IgBLAST (NCBI)

IGHV1-46*01, IGHD3-10*01, & IGHJ4*02

Plasmid Sequences

Plasmids are available upon request as subject to a standard material transfer agreement.

Supplementary references

1 Wang, X. X., Cho, Y. K. & Shusta, E. V. Mining a yeast library for brain endothelial cell-binding antibodies. *Nature methods* **4**, 143-145, doi:10.1038/nmeth993 (2007).

2 Tillotson, B. J., Cho, Y. K. & Shusta, E. V. Cells and cell lysates: a direct approach for engineering antibodies against membrane proteins using yeast surface display. *Methods* **60**, 27-37, doi:10.1016/j.ymeth.2012.03.010 (2013).

Supplementary Figure 1

a

Positive Screening Rounds Against 22 GSC	1	2	3	4	5	6	7	8	9
Total no. yeast applied	5×10^{9}	2×10^{8}	5×10^{7}	5×10^{7}	5×10^{7}	5×10^{7}	5×10^{7}	5×10^{7}	5×10^{7}
Yeast density (yeast/cm^2)	4×10^{7}	4×10^{6}	2×10^{6}	2×10^{6}	2×10^{6}	2×10^{6}	2×10^{6}	2×10^{6}	2×10^{6}
Surface area (cm^2)	126	50.2	25.1	25.1	25.1	25.1	25.1	25.1	25.1
No. recovered yeast	ND	2.58×10^{6}	4.28×10^{5}	9.05×10^{5}	ND	2.58×10^{6}	7.8×10^{6}	5.86×10^{6}	6.26×10^{6}
Recovery percentage	ND	1.3	0.86	1.8	ND	5.2	16	12	13
No. different binders/analyzed yeast	ND	ND	ND	ND	ND	26/110	ND	ND	15/201

b

Negative Screening Rounds Against Co-Culture	S.6	S.7	S.8	S.9
Total no. yeast applied	5×10^{6}	5×10^{6}	5×10^{6}	5×10^{6}
Yeast density (yeast/cm^2)	2×10^{5}	2×10^{5}	2×10^{5}	2×10^{5}
Surface area (cm^2)	25.1	25.1	25.1	25.1
No. depleted yeast	4.77×10^{6}	4.62×10^{6}	4.70×10^{6}	4.66×10^{6}
Depletion percentage	95	93	94	93
No. different binders/analyzed yeast	34/81	19/41	12/41	11/41

c

Re-Positive Screening Against 22 GSC	RS.6
Total no. yeast applied	5×10^{7}
Yeast density (yeast/cm^2)	2×10^{6}
Surface area (cm^2)	25.1
No. recovered yeast	2.62×10^{7}
Recovery percentage	52
No. different binders/analyzed yeast	27/83

d

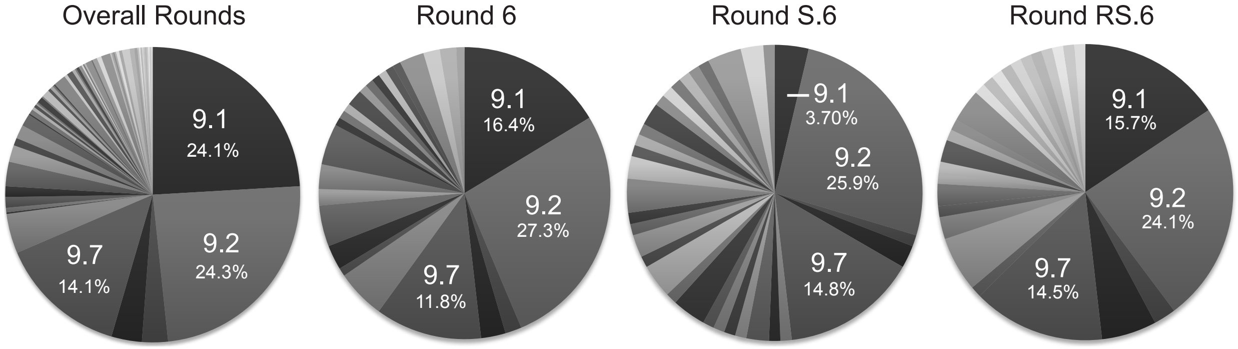

e

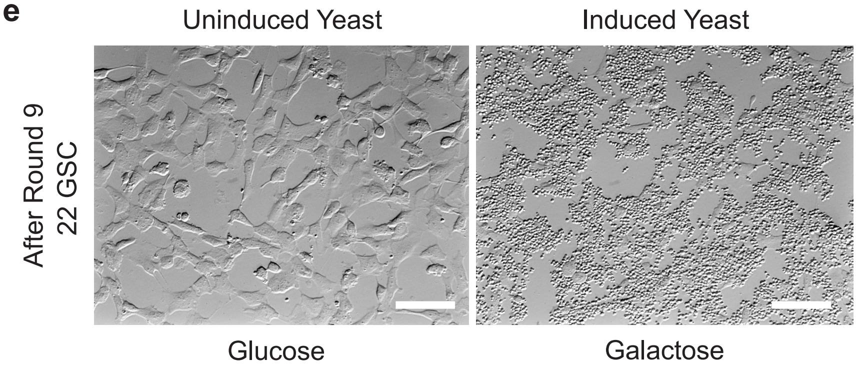

Supplementary Figure 2

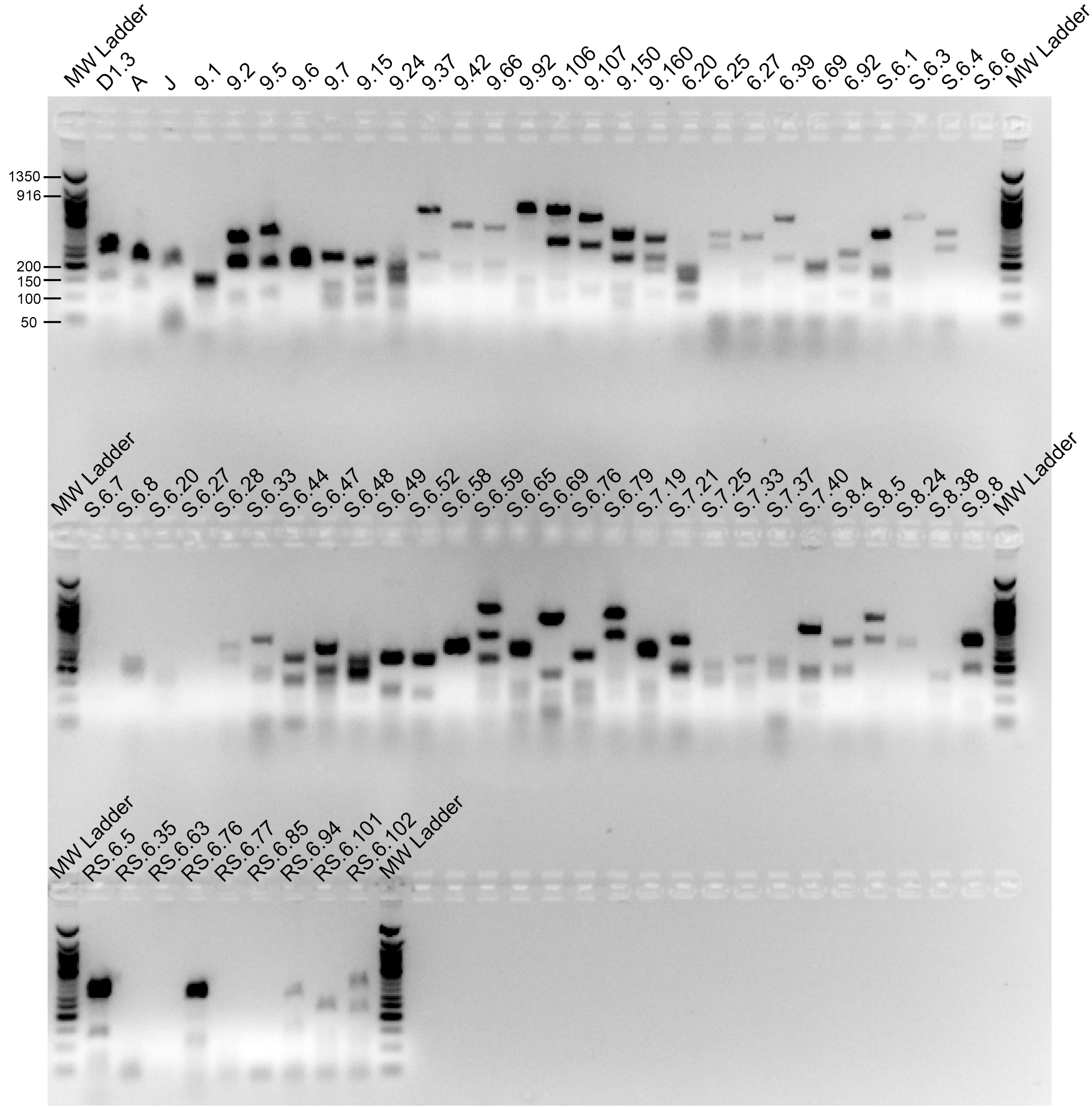

Supplementary Figure 3

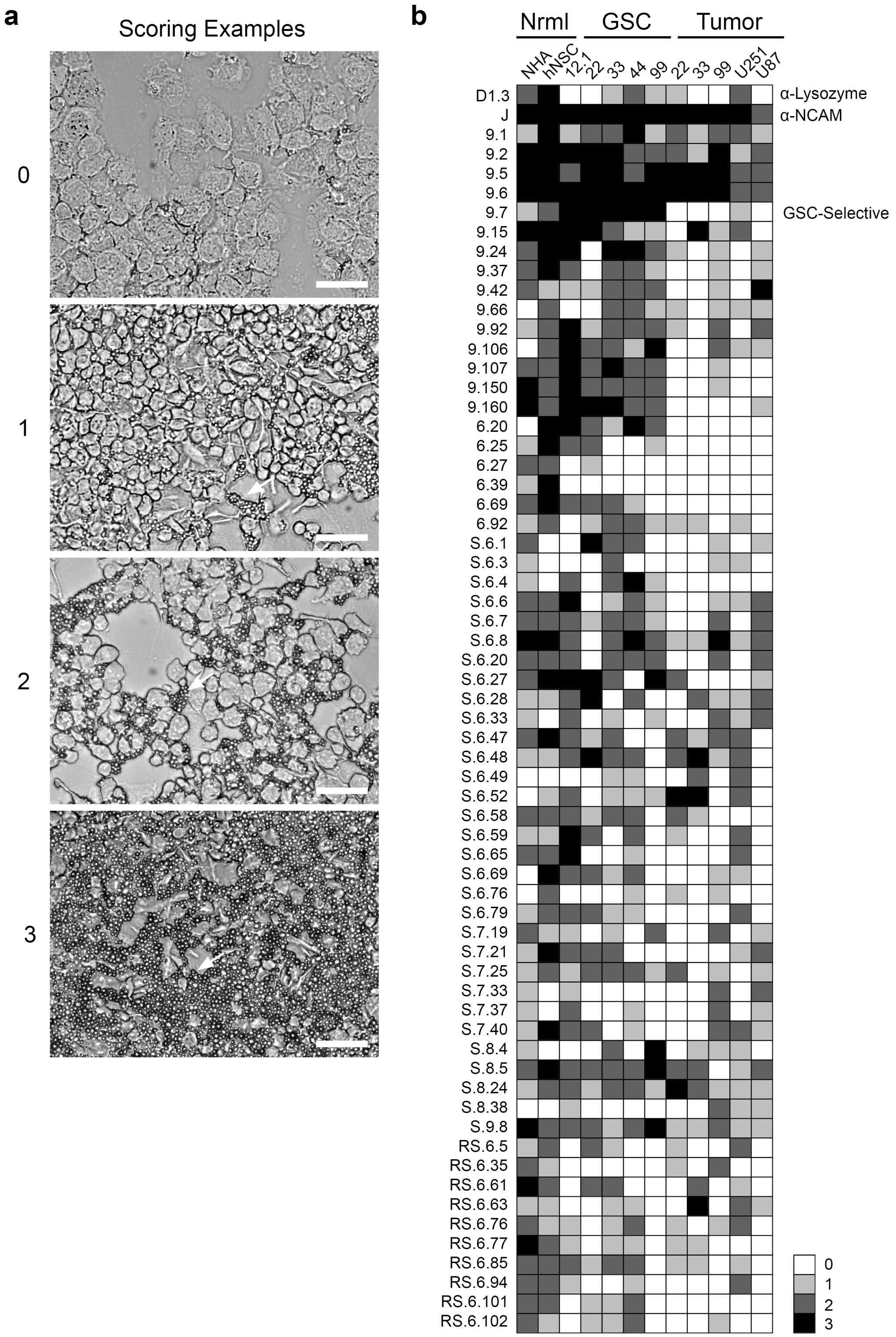

Supplementary Figure 4

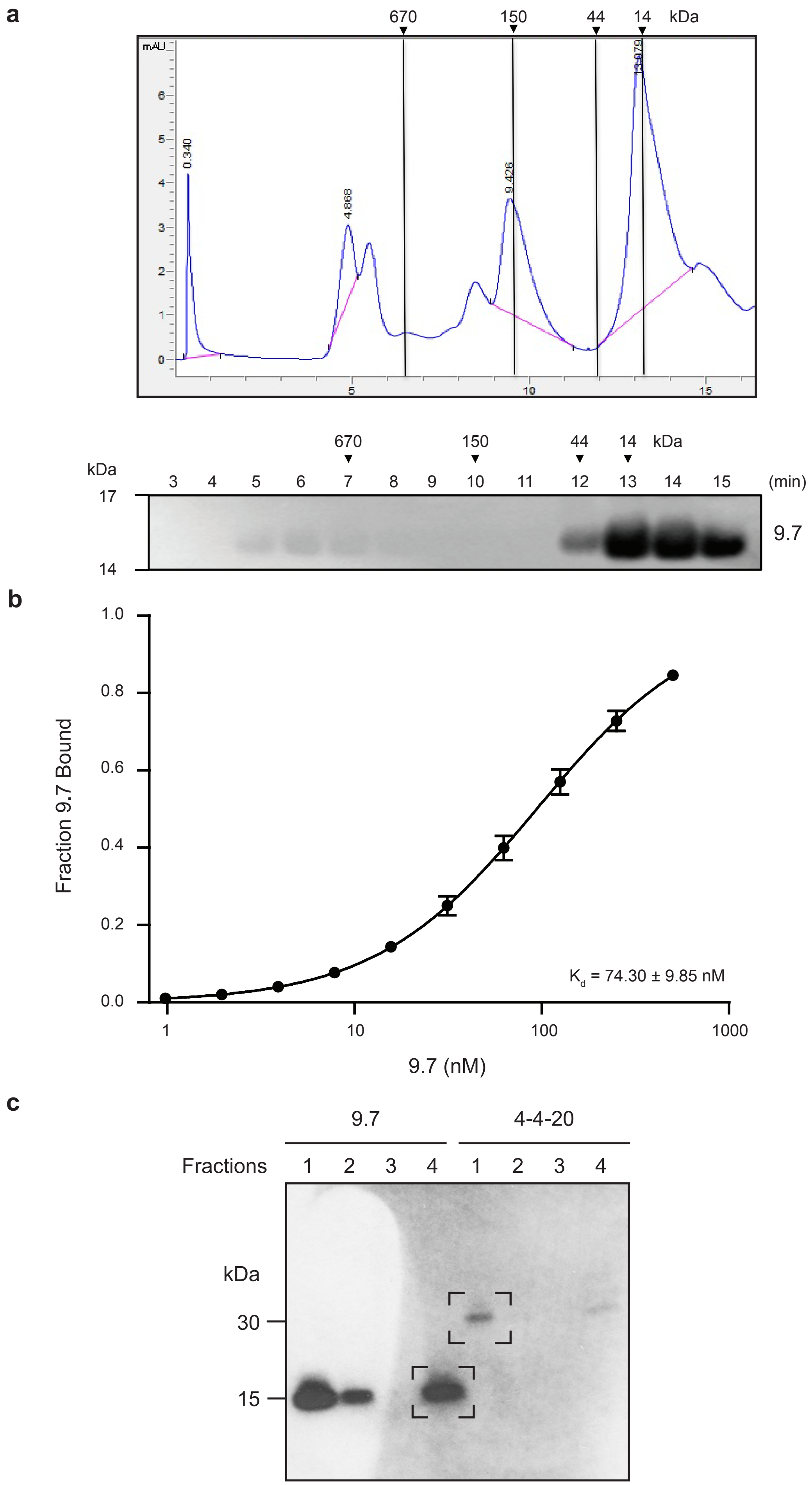

Supplementary Figure 5

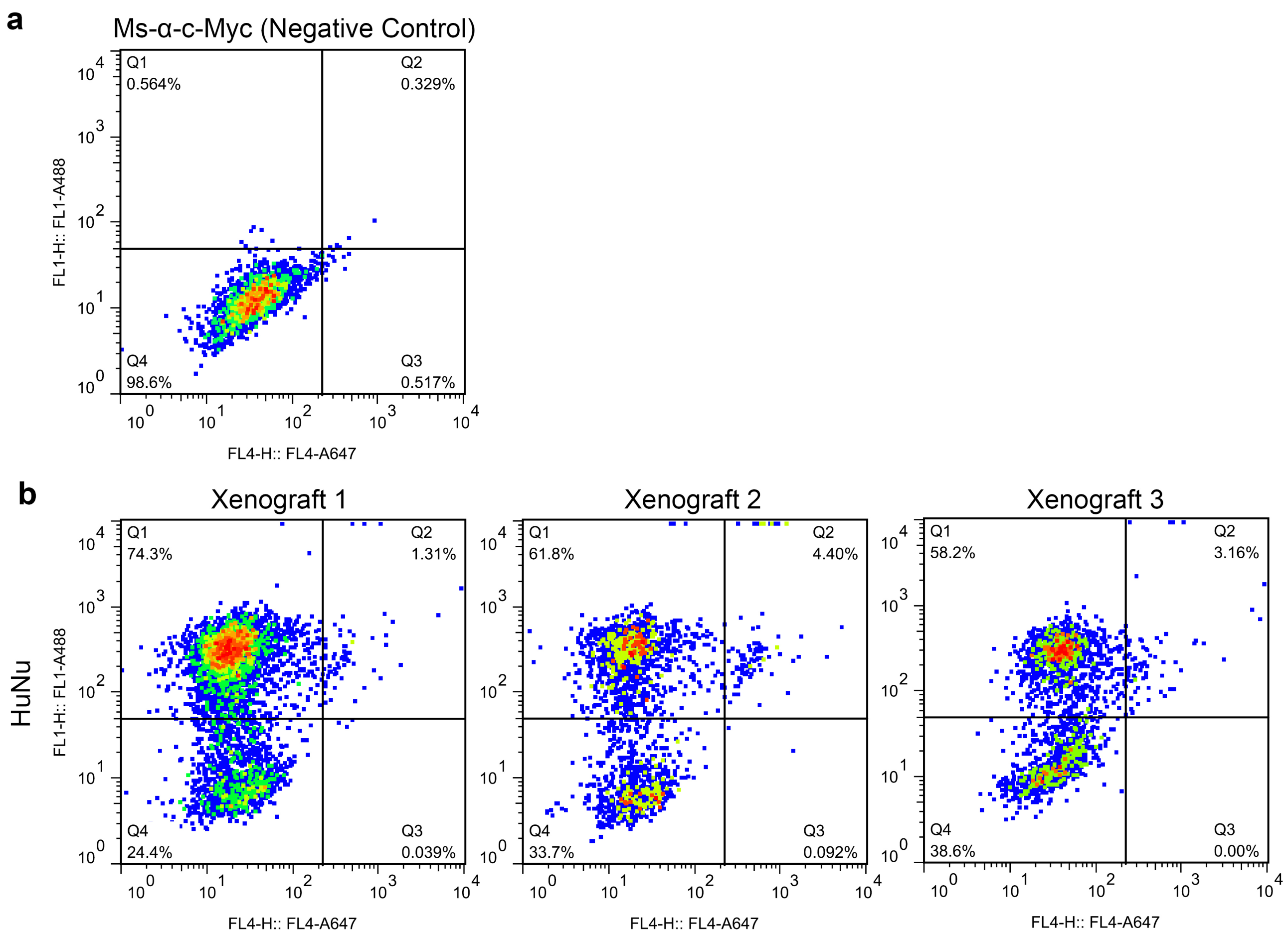

Supplementary Figure 6

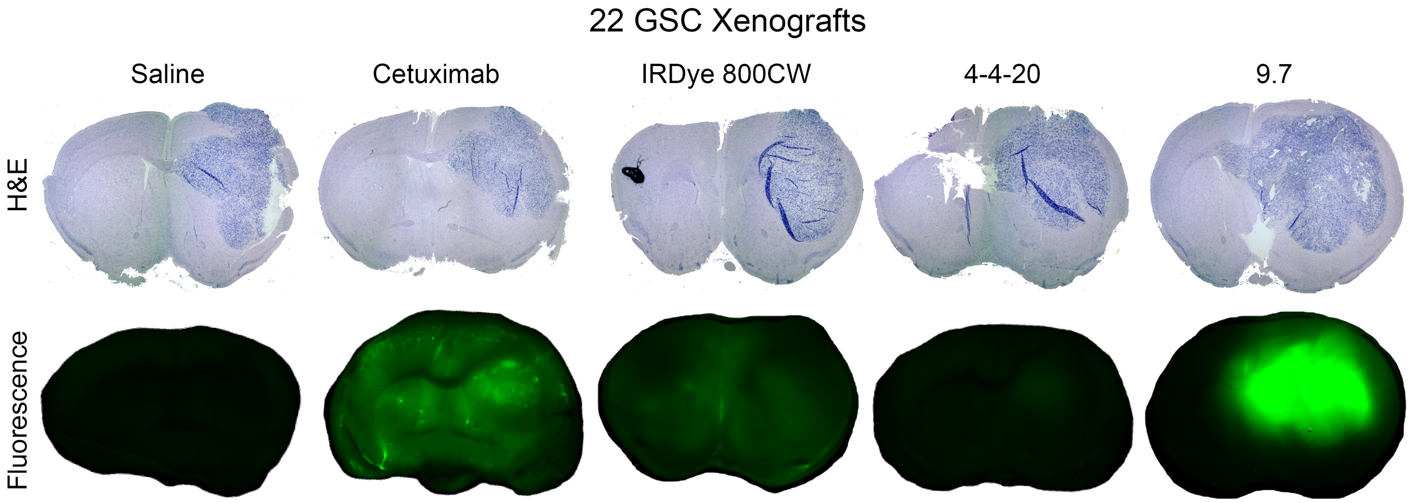

Supplementary Table 1. Yeast scFv clone discovery counts.

#	Clone ID	Round 6	Round 9	Round S.6	Round S.7	Round S.8	Round S.9	Round RS.6	Total
1	9.1	18	92	3	2	6	10	13	144
2	9.2	30	42	21	12	11	9	20	145
3	9.5	2	10	1	0	0	2	2	17
4	9.6	3	4	2	1	3	2	5	20
5	9.7	13	23	12	4	11	9	12	84
6	9.15	6	13	1	0	2	4	0	26
7	9.24	0	1	0	0	0	0	0	1
8	9.37	0	3	0	0	0	0	0	3
9	9.42	1	3	0	2	0	1	0	7
10	9.66	3	1	1	1	0	1	0	7
11	9.92	5	4	2	1	2	1	1	16
12	9.106	2	2	1	1	0	0	5	11
13	9.107	0	1	1	2	1	0	0	5
14	9.150	3	1	1	2	0	0	2	9
15	9.160	5	1	1	0	0	0	1	8
16	6.20	1	0	0	0	0	0	0	1
17	6.25	1	0	0	0	0	0	2	3
18	6.27	1	0	0	0	0	0	1	2
19	6.39	2	0	0	0	0	0	0	2
20	6.69	1	0	0	0	0	0	0	1
21	6.92	1	0	0	0	0	0	0	1
22	S.6.1	1	0	3	0	0	0	0	4
23	S.6.3	0	0	1	0	0	0	0	1
24	S.6.4	1	0	3	0	0	0	1	5
25	S.6.6	1	0	1	0	0	0	0	2
26	S.6.7	0	0	2	0	0	0	0	2
27	S.6.8	0	0	1	0	0	0	0	1
28	S.6.20	0	0	1	0	0	0	0	1
29	S.6.27	0	0	3	5	0	0	0	8
30	S.6.28	0	0	2	0	0	0	0	2
31	S.6.33	1	0	1	0	0	0	2	4
32	S.6.47	0	0	1	0	0	0	1	2
33	S.6.48	0	0	1	0	0	0	0	1
34	S.6.49	0	0	2	0	0	0	0	2
35	S.6.52	0	0	1	0	0	0	1	2
36	S.6.58	0	0	1	0	0	0	0	1
37	S.6.59	0	0	1	0	0	0	0	1
38	S.6.65	0	0	1	0	0	0	0	1
39	S.6.69	0	0	1	0	0	0	3	4
40	S.6.76	0	0	1	0	0	0	0	1
41	S.6.79	0	0	3	0	0	0	0	3
42	S.7.19	0	0	2	1	0	0	0	3
43	S.7.21	3	0	1	1	0	0	0	6
44	S.7.25	0	0	0	1	0	0	0	1
45	S.7.33	0	0	0	1	0	0	0	1
46	S.7.37	0	0	0	1	0	0	0	1
47	S.7.40	0	0	0	1	0	0	0	1
48	S.8.4	0	0	0	0	1	0	1	2
49	S.8.5	0	0	0	1	1	0	0	2
50	S.8.24	2	0	0	1	1	1	0	5
51	S.8.38	2	0	0	0	1	0	0	3
52	S.9.8	1	0	0	0	0	1	0	2
53	RS.6.5	0	0	0	0	0	0	1	1
54	RS.6.35	0	0	0	0	0	0	1	1
55	RS.6.61	0	0	0	0	0	0	1	1
56	RS.6.63	0	0	0	0	0	0	1	1
57	RS.6.76	0	0	0	0	0	0	1	1
58	RS.6.77	0	0	0	0	0	0	1	1
59	RS.6.85	0	0	0	0	0	0	1	1
60	RS.6.94	0	0	0	0	0	0	1	1
61	RS.6.101	0	0	0	0	0	0	1	1
62	RS.6.102	0	0	0	0	0	0	1	1
Unique Clones		26	15	34	19	12	11	27	62
Total Analyzed		110	201	81	41	41	41	83	598
